# What Is Threatening the Effectiveness of Insecticide-Treated Bednets? A Case-Control Study of Environmental, Behavioral, and Physical Factors Associated with Prevention Failure

**DOI:** 10.1371/journal.pone.0132778

**Published:** 2015-07-14

**Authors:** Andrew A. Obala, Judith Nekesa Mangeni, Alyssa Platt, Daniel Aswa, Lucy Abel, Jane Namae, Wendy Prudhomme O'Meara

**Affiliations:** 1 College of Health Sciences, Moi University, Eldoret, Kenya; 2 Duke Global Health Institute, Durham, North Carolina, United States of America; 3 Academic Model Providing Access to Healthcare, Eldoret, Kenya; 4 Webuye Health and Demographic Surveillance System, Moi University, Eldoret, Kenya; 5 Department of Medicine, Duke University, Durham, North Carolina, United States of America; Institut Pasteur, FRANCE

## Abstract

**Background:**

Insecticide-treated nets are the cornerstone of global malaria control and have been shown to reduce malaria morbidity by 50–60%. However, some areas are experiencing a resurgence in malaria following successful control. We describe an efficacy decay framework to understand why high malaria burden persists even under high ITN coverage in a community in western Kenya.

**Methods:**

We enrolled 442 children hospitalized with malaria and paired them with age, time, village and gender-matched controls. We completed comprehensive household and neighborhood assessments including entomological surveillance. The indicators are grouped into five domains in an efficacy decay framework: ITN ownership, compliance, physical integrity, vector susceptibility and facilitating factors. After variable selection, case-control data were analyzed using conditional logistic regression models and mosquito data were analyzed using negative binomial regression. Predictive margins were calculated from logistic regression models.

**Results:**

Measures of ITN coverage and physical integrity were not correlated with hospitalized malaria in our study. However, consistent ITN use (Adjusted Odds Ratio (AOR) = 0.23, 95%CI: 0.12–0.43), presence of nearby larval sites (AOR = 1.137, 95%CI: 1.02–1.27), and specific types of crops (AOR (grains) = 0.446, 95%CI: 0.24–0.82) were significantly correlated with malaria amongst children who owned an ITN. The odds of hospitalization for febrile malaria nearly tripled when one other household member had symptomatic malaria infection (AOR–2.76, 95%CI:1.83–4.18). Overall, perfect household adherence could reduce the probability of hospitalization for malaria to less than 30% (95%CI:0.12–0.46) and adjusting environmental factors such as elimination of larval sites and growing grains nearby could reduce the probability of hospitalization for malaria to less than 20% (95%CI:0.04–0.31).

**Conclusion:**

Availability of ITNs is not the bottleneck for malaria prevention in this community. Behavior change interventions to improve compliance and environmental management of mosquito breeding habitats may greatly enhance ITN efficacy. A better understanding of the relationship between agriculture and mosquito survival and feeding success is needed.

## Introduction

Across the malaria endemic world, coverage with insecticide treated bednets (ITNs) has increased nearly 7-fold in the last 10 years[1]. This has led to considerable reductions in morbidity and mortality from malaria. However, evidence is emerging that malaria control has not been uniformly successful and, in some cases, initial gains may be short-lived[2–7]. Areas where malaria transmission remains impervious to control efforts and areas with resurgence in malaria following initial success hold important lessons for malaria elimination efforts that cannot be ignored.

In Kenya, malaria has historically been the leading cause of child morbidity and mortality. In the last 10 years, ownership of insecticide-treated bednets (ITNs) has increased from 6% to 68%[1, 8]. Artemisinin combination therapy (ACT) has been available at no cost to patients in public facilities since 2006 and available at highly subsidized prices in the retail sector since 2010. Following these investments, reductions in the burden of disease and declining mosquito populations have been documented[9–11]. Recent evidence suggests that in some areas malaria remains low and continues to decline, but in other regions it remains stubbornly high or has started to rise again[4, 5, 7].

Bungoma East is an example of an area where malaria has not declined in proportion to the magnitude of control efforts. ITN coverage in Bungoma East sub-county has increased from 25% [12] to 67%, and in some villages as high as 95% (Webuye HDSS, *pers*. *comm*.) in the last few years. Despite high coverage with ITNs, malaria infection and morbidity remain high. This observed difference between expected effectiveness of an intervention and that achieved in actual implementation outside of controlled trails has been called the ‘efficacy decay’.

**Fig 1:**
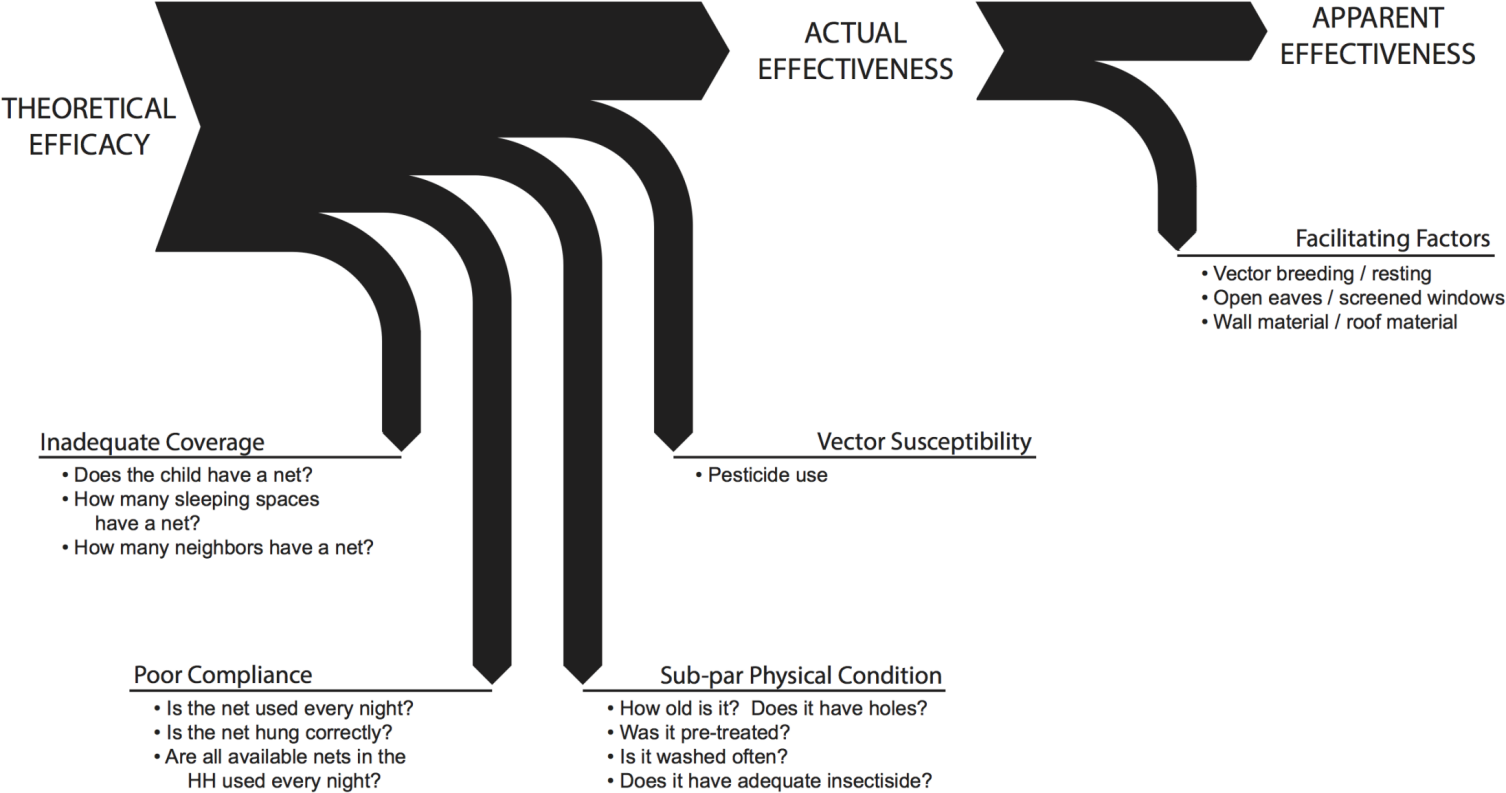
Diagram of the efficacy decay of prevention. Effectiveness of ITNs is threatened by five domains: inadequate coverage, poor compliance, poor physical condition and reduced vector susceptibility. In addition, facilitating factors can further reduce the apparent effectiveness measured under real-world conditions.

Here we deconstruct the ‘efficacy decay’ of ITNs in terms of a series of interdependent domains. ITNs have an efficacy of 50–60% percent in preventing disease in randomized controlled trials[13]. However, in order for ITNs to be effective at the individual level, a household must own an ITN, use it consistently, and the ITN must be in good physical condition (few or no holes, adequate levels of insecticide). In addition, the vector population must be susceptible to the insecticide and must bite during the hours when individuals are protected by the ITN. Reduction in any of these factors may lead to a decline in ITN effectiveness. Presence of favorable facilitating factors may lead to further reduction in apparent effectiveness. Together, these five domains contribute to the *efficacy decay of prevention* (Fig 1).

Single steps along the efficacy decay have been isolated, explored and quantified, but what has not been well described is the relative contribution of these domains to the total reduction of ITN efficacy resulting in low apparent effectiveness. We employed case-control methodology and analyze our data within the efficacy decay framework in order to parse reasons for continuing high morbidity in areas where bednet coverage is high.

## Methods

### Study site

Bungoma East sub-county (formerly Bungoma East district) is located approximately 50 km east of the border with Uganda. Commercial farming of sugarcane is the predominant economic activity and involves use of organophosphate and pyrethroid classes of insecticides. Most families (>60%) live below the poverty line. Malaria transmission is perennial with a seasonal peak following the rains in May-June. Studies in a neighboring district report that 23% of *Anopheles gambiae s*.*l*. are *A*. *arabiensis* and the prevalence of homozygous resistant kdr genotype in *A*. *gamabiae s*.*s*. was 99%. Prior to scale-up of control efforts, entomological inoculation rate (EIR) was estimated to be 29 infectious bites per person per year[14].

Webuye Sub-county Hospital (latitude: 0° 36' 26.244", longitude: 34° 46' 19.056") is the main referral hospital for families in Bungoma East. 400 children are admitted to the ward each month, between half and two-thirds of these are for malaria, depending on the season. There are 12 other public health facilities–nine dispensaries, two health centres, and one sub-sub-county hospital.

The study area is predominantly rural with a small peri-urban center immediately surrounding the hospital. Approximately 10 percent of our study population resides in the peri-urban area.

### Cases and controls

For this study, we compared children hospitalized with moderate to severe malaria to uninfected controls. Children must first become infected, and then some may become symptomatic and treated as outpatients while some may progress further and require hospitalization. Not all of the possible outcomes are represented in the hospitalized group. However, all hospitalized malaria cases must first go from uninfected to infected. Our variables of interest focused on this first step of infection (or failure of prevention). We did not evaluate reasons for progressing from infection to symptomatic and severe disease. Therefore, we refer to our outcome as ‘malaria’, understanding that there are outcomes of infection that are not explicitly present in our definition of cases.

Cases were recruited from the pediatric inpatient ward of Webuye Sub-county Hospital. Children between the ages of 1–10 years with malaria as the primary cause of admission who resided within the study boundaries (six administrative sublocations surrounding the hospital) were eligible for the study. Malaria infection was confirmed using a malaria rapid diagnostic test (RDT, Standard Diagnostics SD Bioline Malaria Ag P.f.(HRPII)) prior to enrollment. Consecutive eligible cases were enrolled until the sample size was met.

Each case was visited at home after they were discharged. Households within the same village, but outside of the search radius for neighbors and larval sites (250 meters) were canvassed to identify an age- and gender-matched healthy control. The control was recruited on the same day that the case household was visited which was a maximum of one week after the date of admission to the hospital. Control children were only enrolled after confirming absence of malaria infection using an RDT and were excluded if they were unwell on the day of recruitment, or had been ill or taken antimalarials in the last one month.

Data were captured on mobile phones running the android operating system using the Open Data Kit platform. Household information was recorded including the number of household members, education and occupation of the household head, housing construction (material of walls, roof and floor, screened windows, open eaves), and agricultural practices including the types of crops grown around the house and the names of all chemical products used on the crops as reported by the family.

### ITN coverage, use and efficacy

Each sleeping space, the members sleeping in the space, and the ITN (if any) for that space was inventoried. Physical integrity of the net was recorded using a 5-point Likert scale from “very good condition” to “very bad condition.” Number and size of holes (categorized by smaller than a coin, larger than a coin but smaller than a hand, and larger than a hand to describe the largest hole), age of each ITN, frequency of washing and whether the net was treated/pre-treated were also recorded. Questions describing the frequency of use, and any circumstances under which the net was not used were asked. The caretaker of the enrolled child was asked to demonstrate how they arranged the net for the child at night and this was scored by the interviewer. A photograph was taken to allow further evaluation of the net condition and use. The net of the enrolled child was collected for testing and replaced with a new ITN. ITNs were tested for adequate insecticide levels using the standardized WHO bioassay[15]. Briefly, 10 susceptible female lab-reared *Anopheles gambiae* mosquitoes were exposed to five different sections of the ITN cut from different faces of the ITN for a total of 50 mosquitoes per ITN. Knockdown rates were measured at 3 minutes and total mortality rate at 24 hours. An untreated control net was also tested in parallel on each day. Where knockdown and mortality rates were 5%—<20%, Abbotts formula was used to calculate an adjusted rate [16]. Nets that achieved less than 80% knockdown or kill rates were defined as substandard.

### Parasite burden

All family members of each case and control child were tested for current and recent malaria infection using HRP2-based RDTs. RDT-positive family members received artemether lumefantrine in an appropriate dose or were referred to the nearest health facility if younger than 6 months old or pregnant.

### Entomological measures

All potential vector breeding sites within a quarter-kilometer radius around the home were mapped and photographed. Each breeding site was dipped to determine the presence of any mosquito larvae (anopheles or culicine). A miniature CDC light trap was placed in the first case and control household enrolled each week. Mosquitoes were collected between 18.30 h and 6.30 h each night for four consecutive nights. This was repeated every other week for two months for a total of 16 nights of collection per house.

### Neighborhood ITN coverage

Previous studies have shown that when approximately 50% of individuals in an area are protected by an ITN, even individuals not sleeping under an ITN enjoy significant indirect protection[17, 18]. Households surrounding the case or control household within a radius of 0.25 km were surveyed to measure the number of ITNs per household and the ratio of ITNs to sleeping spaces.

### Ethical considerations

The study protocol, including all consent procedures, was reviewed and approved by the Moi University Institutional Research and Ethics Committee and Duke University Institutional Review Board. The household head provided written informed consent for participation in the survey. In addition, individual written informed consent was required from each household member, or the parent/guardian in case of minors, before RDT testing. Written documentation of assent was also required for children older than 8 years of age. A waiver of documentation of consent was granted from both institutions for questionnaire administration to *neighbors* of case-control children because the neighbor questionnaire presented no more than minimal risk, collected no identifying information from neighbor’s households, and a written consent form would be the only identifying information linking the individual to the data.

### Sample size and analysis

The primary outcome measure is the odds ratio of hospitalized malaria in those children with and without an ITN which is estimated for a matched case control sample by conditional logistic regression. A sample size of 450 children per group was selected to give 80% power to measure an odds ratio of at least 0.60 for ITN ownership in the cases compared to the controls with 95% confidence. An OR of 0.6 corresponds to 40% protection afforded by ITN ownership.

Due to the number of covariates and the analytic goal of finding the best fitting model for inference on febrile malaria risk, we employed a “best subsets” method of variable selection using the branch-and-bound algorithm[19]. The branch-and-bound algorithm is used to find the best regression model of each size (number of covariates) defined as that with the highest likelihood score (chi-square). We computed Akaike information criteria (AIC) for each selected model of each size. We then selected the model that best combined AIC score with parsimony as the final model. Selection for the febrile malaria outcome was conducted using PROC LOGISTIC in SAS 9.4. The conditional logistic regression was repeated in STATA 13 using the selected variables.

Counts of mosquitoes and fed mosquitoes caught in light traps exhibited significant overdispersion; no mosquitoes were caught in 33% of households, one caught in 12% of households and 2 or more mosquitos were caught in the remaining 55%. Variances of mosquito counts exceeded means, thus we used negative binomial regression models with an offset of log of number of nights during which traps were set in each household. We incorporated the case-control pair as a stratum in a complex survey design framework in order to retain and control for the matched (temporally and geographically) nature of the sample. Due to the reduced sample size of the entomology data (N = 98) and the complex survey design framework, we ran univariate negative binomial regressions for each covariate and outcome, selected any covariate with p≤0.30 and used those covariates in a final regression model. Next we eliminated variables from the multivariable model in a backwards stepwise manner, using a Wald statistic to eliminate variables one-by-one that had p-values of greater than 0.30. All eliminated variables were added to the final regression model, one at a time, to confirm their lack of significance for inference in the final model. Those that tested significant via a Wald test statistic, were added back into the model one at a time until further additions did not produce statistically significant results. All variable selection for mosquito density analysis was conducted using Stata 13.1 software using the svy survey data analysis procedures.

## Results

The study was conducted between May 2013 and July 2014. We enrolled 442 matched case-control pairs. The mean age of index children (case and controls) was 3.5 years. Fifty percent of enrolled children were male (Table 1).

**Table 1:** Characteristics of case/control children and their households.

	Case		Control	
Variable	Mean	95% Confidence Interval	Mean	95% Confidence Interval
**Children and Households**				
Age (years)	3.58	(3.31–3.85)	3.49	(3.24–3.75)
Male	0.50		0.50	
Proportion testing positive (household)	0.20		0.08	
Total persons in household	6.19	(5.98–6.40)	6.17	(5.95–6.38)
Total nets in household	1.86	(1.75–1.97)	1.91	(1.81–2.02)
Sleeping spaces per person	0.45	(0.44–0.46)	0.46	(0.45–0.47)
Household head finished secondary school	0.30		0.28	
**Net Ownership and Coverage**				
***Coverage***				
Proportion with net	0.82		0.81	
Household has at least 1 net	0.90		0.92	
Nets per person	0.31	(0.29–0.33)	0.33	(0.31–0.34)
Net to person ratio of at least 1:2	0.20		0.21	
Nets = Sleeping spaces	0.51		0.53	
Percent of neighbors with at least 1 net	0.92	(0.91–0.93)	0.93	(0.93–0.94)
***Compliance and correct use***				
Percent of neighbors under net	0.79	(0.78–0.81)	0.80	(0.79–0.81)
Used net last night	0.75		0.75	
Used net every day of past week	0.63		0.74	
Ever a time when net is not used	0.26		0.15	
***Physical integrity***				
Net in good condition (index child)	0.55		0.55	
Net pre-treated (index child)	0.97		0.99	
Nets in good condition (per person)	0.44		0.47	
All nets in good condition	0.40		0.45	
Age of net (years)	2.41	(2.24–2.58)	2.34	(2.18–2.50)
Proportion failed bioefficacy test (mortality)	0.11		0.11	
Proportion failed bioefficacy test (knock-down)	0.04		0.03	
Net washed frequently	0.11		0.11	
**Vector Susceptibility**	** **	** **	** **	** **
***Pesticides used***				
Organophosphate	0.13		0.10	
Pyrethroid	0.05		0.06	
Carbamate	0.01		0.00	
Any pesticide	0.16		0.15	
**Facilitating Factors**				
***Types of crops***				
Grains	0.70		0.74	
Sugar cane	0.67		0.64	
Napier grass	0.31		0.29	
Banana	0.73		0.75	
Vegetables	0.56		0.56	
Legumes	0.48		0.45	
Tubers	0.44		0.42	
***Larval sites***				
Total larval sites	2.13	(1.91–2.34)	1.48	(1.29–1.67)
Total sites with larvae	0.41	(0.34–0.49)	0.22	(0.16–0.28)
***Housing construction***				
Open eaves	0.38		0.31	
Thatched roof	0.04		0.05	
Nonporous wall material	0.20		0.19	
N	442		442	

We collected detailed data on four domains relevant to ITN effectiveness as well as potential facilitating factors (Fig 1, Table 1). First, we looked at net ownership and coverage within the household and amongst neighbors. Then, we explored compliance and correct net use. Physical condition of all household nets and bio-efficacy of the case/control child net was evaluated. We touched on vector susceptibility to ITNs by testing the role of pesticides (such as organophosphates, pyrethroid, and carbamates) used on crops on protection offered by nets. Finally, we captured data on potential facilitating factors including housing construction, nearby agriculture, and nearby larval sites. Unadjusted odds ratios for every tested variable are provided in the supplementary information (S1 Table).

### Is coverage enough?

Eighty percent of index children had an ITN for their sleeping space. There was no difference in ITN ownership between case and control children. The odds of being a case was not significantly different between children who had or did not have an ITN for their sleeping space.

The World Health Organization has identified indicators for universal or adequate coverage of the population with insecticide treated nets. In order of increasing stringency these are; 1) household has at least one net, 2) household has a net for each sleeping space and 3) the household has at least one net for every two people. Although approximately 90% of households had at least one net, only 50% of households had a net for each sleeping space and only 20% had a ratio of nets to people of at least 1 to 2. We tested whether children in households meeting these coverage criteria had lower odds of malaria and found that there was no difference in the odds of malaria between households meeting or not meeting these three criteria (Table 2).

**Table 2:** Is net coverage enough?

N = 876	Percent		Odds Ratio	95% Conf. Interval		P>z
	Case	Control				
Index child has net	82.0%	81.3%	1.102	0.774	1.567	0.59
Any net in household	89.5%	92.0%	0.744	0.471	1.176	0.206
Ratio of nets to household members is at least 1:2	20.1%	21.2%	0.944	0.676	1.318	0.734
Net for every sleeping space	50.5%	53.4%	0.886	0.678	1.158	0.376

### What puts children at risk?

We used as our initial sample all index children and their homes, regardless of net ownership. The results of the final model are shown in Table 3. Recent travel to another malaria endemic area (Adjusted Odds Ratio (AOR) = 3.16, 95% CI:1.08–9.22) or residing in a household with other malaria-infected individuals, either symptomatic (AOR = 1.89, 95%CI:1.37–2.62) or asymptomatic (AOR = 2.59, 95% CI:1.92–3.50), greatly increased the odds of infection. Nearby breeding sites and the presence of larvae in those sites were also important risk factors. Each additional larval site identified within 250 meters of the home increased the odds of infection by 17% (AOR = 1.17, 95%CI:1.07–1.28). The effect was nearly twice that for sites where larvae were present (AOR = 1.33, 95%CI:1.02–1.74). Housing construction, specifically open eaves and cement walls, increased the odds of infection, although these relationships did not quite reach statistical significance. Grains grown nearby significantly reduced the odds of infection (AOR = 0.55, 95%CI:0.33–0.92). In agreement with the results above, ITN ownership by the index child was not identified as an important factor.

**Table 3:** Conditional logistic regression of variables related to malaria infection amongst all enrolled children and all children with a net for their sleeping space.

		All children (n = 872)		Children with ITN (n = 594)	
		Unadjusted OR	Adj. OR	Unadjusted OR	Adj. OR
	Domain	(95% CI)	(95% CI)	(95% CI)	(95% CI)
Total RDT positive, no symptoms	*Facilitating factor*	**1.69**	**1.894**	**1.907**	**2.149**
		**(1.290–2.215)**	**(1.368–2.623)**	**(1.327–2.742)**	**(1.336–3.458)**
Total RDT positive, some symptoms	*Facilitating factor*	**2.379**	**2.593**	**2.446**	**2.764**
		**(1.824–3.102)**	**(1.923–3.497)**	**(1.720–3.480)**	**(1.826–4.183)**
Enrolled child: Net used every day (7 days/week)	*Compliance*	**-**	**-**	**0.286**	**0.228**
		**-**	**-**	**(0.169–0.482)**	**(0.122–0.426)**
Total persons NOT under net last night	*Compliance*	**-**	**-**	1.056	**0.834**
		**-**	**-**	(0.971–1.148)	**(0.739–0.942)**
Net for every sleeping space	*Coverage*	0.886	1.165	**-**	**-**
		(0.678–1.318)	(0.826–1.642)	**-**	**-**
All nets in good condition	*Physical integrity*	0.814	0.826	**-**	**-**
		(0.618–1.072)	(0.593–1.151)	**-**	**-**
Open eaves	*Facilitating factor*	**1.403**	1.342	**-**	**-**
		**(1.047–1.879)**	(0.953–1.890)	**-**	**-**
Wall material non-porous (bricks, blocks, cement, stone)	*Facilitating factor*	1.133	1.625	**-**	**-**
		(0.759–1.692)	(0.989–2.668)	**-**	**-**
Travelled to malaria endemic region	*Compliance*	**2.833**	**3.158**	**-**	**-**
		**(1.117–7.186)**	**(1.082–9.219)**	**-**	**-**
Total persons in household		1.005	0.930	**-**	**-**
		(0.946–1.067)	(0.857–1.010)	**-**	**-**
Grains	*Facilitating factor*	**0.643**	**0.550**	**0.523**	**0.446**
		**(0.423–0.977)**	**(0.329–0.920)**	**(0.316–0.866)**	**(0.241–0.824)**
Vegetables	*Facilitating factor*	0.971	1.108	**-**	**-**
		(0.692–1.362)	(0.735–1.669)	**-**	**-**
Legumes	*Facilitating factor*	1.181	1.328	**-**	**-**
		(0.862–1.616)	(0.891–1.980)	**-**	**-**
Total larval sites	*Facilitating factor*	**1.202**	**1.171**	**1.178**	**1.137**
		**(1.112–1.298)**	**(1.067–1.284)**	**(1.081–1.284)**	**(1.020–1.269)**
Total sites with larvae	*Facilitating factor*	**1.518**	**1.334**	**1.469**	**1.426**
		**(1.220–1.888)**	**(1.024–1.739)**	**(1.148–1.880)**	**(1.045–1.945)**
Pesticide	*Vector susceptibility*	1.143	1.147	1.25	1.715
		(0.779–1.677)	(0.706–1.863)	(0.785–1.990)	(0.948–3.105)

Statistically significant parameter estimates in **bold**

### Beyond net ownership

We next focused on a subset of children who had an ITN for their sleeping space so we could explore factors related to correct usage and condition of the net (Table 3). There were 297 matched cases and controls where both the case and control child had a net (n = 594).

A child who used the net every night during the last week had significantly lower odds of malaria infection (AOR = 0.23, 95%CI:0.12–0.43). The odds of being infected increased more than 2-fold for each additional infected asymptomatic household member and nearly three-fold for each additional symptomatic infected member (AOR asymptomatic = 2.15, 95%CI:1.33–3.46, AOR symptomatic = 2.76, 95%CI:1.83–4.18). Odds of malaria for an individual child declined with increasing numbers of household members *not* protected by a net (AOR = 0.83, 95%CI 0.74–0.94), perhaps indicating that individual risk from an infected mosquito goes down as the number of possible targets goes up, particularly if other targets are exposed.

Agriculture practices around the home were significantly correlated with malaria. As above, growing grains like maize, wheat, millet or sorghum reduced the odds of malaria (AOR = 0.45, 95%CI:0.24–0.82). However, using pesticides increased the odds of malaria, although this relationship did not reach significance at the 95% level (AOR = 1.72, p = 0.078). Agriculture practices may be related to local breeding sites, which were again significantly correlated with increased odds of malaria.

### Entomological risk factors

We captured mosquitoes using CDC light traps in matched case-control household pairs for an average of 15 nights per household (Table 4). Forty-nine case-control pairs were enrolled in entomological surveillance giving a total of 1566 nights of collection across the study area (Table 4). We captured a total of 1061 female anopheles mosquitoes; twenty-one percent of trapped female *Anopheles* had fed. The number of malaria vectors trapped per household was highly over-dispersed; 32.6% of households had no mosquitoes trapped at all.

**Table 4:** Entomological surveillance.

Variables	Case	Control
Number of households	49	49
Number of nights of trapping	15.3	15.3
Number of households where mosquitoes were caught^a^	37 (75.5)	29 (59.2)
Mean number of mosquitoes (per nights of trapping) caught in households with mosquitoes^b^	0.75 (0.51–0.99)	0.57 (0.36–0.78)
Mean number of fed mosquitoes (per nights of trapping)	0.17 (0.055–0.29)	0.09 (0.006–0.17)

^a^N (%)

^b^Rate (95% CI)

There was no difference in the rate of mosquitoes or blood fed mosquitoes trapped in case versus control household (Table 5, Adj.IRR = 1.04, 95%CI:0.48–2.22 and Adj.IRR = 1.19, 95%CI:0.53–2.70, respectively). As the ratio of ITNs in good condition to total household members increased, the rate of fed mosquitoes trapped decreased. Each additional larval site within 500m increased the rate of mosquitoes trapped by >35% (Adj.IRR = 1.36 95% CI: 1.118–1.658).

**Table 5:** Multivariable negative binomial regressions of anopheles mosquito counts.

	(1)	(2)
Variables	Total anopheles	Total fed anopheles
Case child	1.035	1.192
	(0.483–2.218)	(0.527–2.697)
Total nets in household (per person)	**0.203**	
	**(0.0519–0.793)**	
Total nets in good condition (per person)		0.423
		(0.179–1.003)
Total persons in household		1.105
		(0.969–1.259)
Windows Protected	1.826	
	(0.873–3.816)	
Grains	**3.221**	**3.084**
	**(1.658–6.258)**	**(1.209–7.866)**
Vegetables	**2.108**	**2.595**
	**(1.164–3.819)**	**(1.341–5.022)**
Legumes	**3.459**	**2.725**
	**(1.578–7.584)**	**(1.090–6.813)**
Tubers	**0.333**	**0.323**
	**(0.185–0.596)**	**(0.165–0.634)**
Pesticide	0.288	**0.135**
	(0.0806–1.027)	**(0.0406–0.449)**
Total larval sites	**1.361**	**1.266**
	**(1.118–1.658)**	**(1.040–1.542)**
Constant	**0.107**	**0.0122**
	**(0.0457–0.253)**	**(0.00285–0.0522)**
Alpha	2.091	2.325
	(1.611–2.713)	(1.479–3.653)
Observations	98	98

Similar to the case control analysis, negative binomial regression showed that certain crops were associated with higher numbers of mosquitoes and fed mosquitoes that were trapped (grains, legumes), but using pesticides decreased the density of fed mosquitoes.

### Efficacy decay of prevention

To compare the relative contribution of ITN coverage, adherence, physical integrity and facilitating factors to the odds of malaria, the selected variables from Table 3 were grouped by domain and the predictive margins were estimated from the multivariate models (Table 6). ITN ownership was not identified as important at the individual, household or neighborhood level and therefore had no impact on the predicted probability of malaria. Adherence to daily ITN use was strongly correlated with malaria and the probability of being hospitalized with malaria is 28% amongst those with perfect adherence living in a household with perfect adherence compared to 58% amongst children who do not use an ITN every day in a household with average adherence. However, removing all facilitating factors such as larval sites and changing agriculture practices gives a probability of malaria of 18% under conditions of *average* adherence. Combining perfect adherence with environmental management reduces the probability of malaria by only an additional 1%. Even without an ITN, removing facilitating factors would reduce the probability of malaria to less than 50%.

**Table 6:** Predictive margins. Probability of malaria for different values of specific covariates by domain. Each scenario holds all other model covariates at their mean value.

**Children with ITNs**			
***Scenario***	**Probability**	**95% Confidence Interval**	
**Poor individual adherence:** ITN not used every day	0.58	0.44	0.71
**Perfect individual adherence:** ITN used every day	0.24	0.08	0.40
**Perfect household adherence**: ITN used everyday, all household members used ITN last night	0.29	0.12	0.46
**Vector susceptibility:** Pesticides not used	0.27	0.11	0.42
**Facilitating factors:** No nearby larval sites, grains grown nearby	0.18	0.04	0.31
**Facilitating factors x adherence:** No nearby larval sites, grains grown, ITN used every day and all household members used ITN last night	0.17	0.04	0.31
			
**All children (80% with an ITN)**	** **	** **	** **
***Scenario***	**Probability**	**95% Confidence Interval**	
**Poor household adherence:** Recently traveled	0.83	0.64	1.02
**Perfect household adherence:** Every sleeping spaces has a net and no recent travel	0.59	0.37	0.82
**Vector susceptibility:** No pesticide use	0.60	0.41	0.79
**Facilitating factors:** Closed eaves, mud walls, no nearby larval sites, grains grown, no vegetables or legumes grown	0.45	0.27	0.64

## Discussion

When high ITN coverage fails to produce the expected reduction in malaria burden, we must assume a breakdown in other essential aspects of prevention. In our study area, high malaria burden persists in the context of good ITN coverage. In our study, the overall prevalence of *P*. *falciparum* infection in children less than 10 years of age was 22% and reached 50% in the rainy season but ITN coverage of their sleeping spaces was 75%, indicating moderate to high seasonal transmission in the context of good ITN coverage. We investigated factors contributing to the lower than expected impact of ITNs across five key domains–ITN ownership, compliance, physical condition, vector susceptibility, and facilitating factors–and quantified their relative contribution to malaria morbidity in children. The key barriers to prevention in our study area are individual compliance with net use and environmental factors that contribute to continued vector success in feeding and transmission.

Surveillance of ITN programs has generally focused on a limited number of metrics that describe ITN coverage, such as the proportion of households owning an ITN, the ratio of ITNs to sleeping spaces within a household, or the proportion of household members who slept under an ITN the previous night. We note in our study that >80% of index children had an ITN and ninety percent of households had at least one. World Health Organization benchmarks for ITN coverage did not predict malaria disease amongst children. We suggest that, in this context, continuing to focus on ITN distribution alone may not produce further reduction in malaria burden and measuring success based on such indicators may be misleading. The lack of importance of net condition and net bioefficacy also indicate that additional ITNs or replacement of existing ITNs with new ones are likely not the optimum strategy in this context.

Amongst children with an ITN, the most important factor in reducing infection was consistent use of the ITN. We asked whether a child used the net last night, how many nights in the last week the net was used, and whether there was ever a time that the child did not use the net. We also evaluated how likely it was that a child used the net consistently based on whether the net had a permanent hanging position or had to be hung each night. The best single measure of consistent net use related to reducing the odds of malaria was the number of nights the child used the net in the last seven. The importance of consistent ITN use was further highlighted by the increased odds of malaria in children who travelled away from the home in the last month. While away from home, children may be less likely to use a net, for example in a temporary sleeping space. However, they may also be at higher risk if they are exposed to higher transmission intensity or new parasite immuno-types.

In this community, as is common in much of sub-Saharan Africa, dwellings are interspersed with small-scale agriculture. Unlike areas with commercial-scale farming, mosquitoes exposed to agricultural pesticides in small-scale farming are the same mosquitoes seeking bloodmeals from adjacent homes. Local agriculture and pesticide use could influence mosquito populations and susceptibility to ITNs, and that these same mosquitoes might be responsible for transmission to nearby human hosts. Other studies have shown a relationship between agriculture intensity, pesticide use, and insecticide resistance of malaria vectors[20–27]. In our study area, the type of crops grown near the home were strongly correlated to malaria and to mosquito density. Pesticide use was marginally associated with increased odds of malaria amongst children with an ITN. The fact that the pesticide effect was specific to children with an ITN suggests that it could be related to reduced sensitivity to insecticides used in the ITN, although this requires more detailed investigation to confirm. Although the reported use of pyrethroids in crop maintenance was low, phenotypic resistance to multiple classes of insecticides mediated through increased insecticide metabolism has been reported[23, 28].

The apparent protective effect of growing grains may reflect the absence of other types of crops or use of an irrigation scheme that is less conducive to creating breeding habitats[29]. The results from the mosquito abundance analysis seem to contradict those of the case-control analysis; in the mosquito models, growing grains was correlated with higher mosquito density, along with growing vegetables and legumes. The most commonly grown grain in this community is maize, the pollen of which is a food source for larvae[30]. This may account for its contribution to mosquito density in our model. Lower rates of mosquito trappings were observed in households where tubers were grown nearby. Interestingly, a positive association between malaria morbidity and growing beans or sweet potatoes and an inverse association with millet and sorghum farming was reported from Uganda[31]. The significance and specific mechanisms of these relationships are unknown and require further investigation. Although we controlled for pesticide use in our analysis, it has been observed in some studies that the presence in larval habitats of herbicides, fungicides, fertilizers and even plant material containing natural xenobiotics can modify mosquito susceptibility to pyrethroids through modulation of their detoxification pathways[24, 32–35]. It is possible that such chemicals are used differentially on specific crops and we have measured the impact of this heterogeneity on mosquitoes and malaria transmission.

The consistent importance of both active and suspected mosquito breeding habitats in malaria infection and mosquito density underscore the need for integrated vector control. Larval site reduction and environmental management may be the key to maximizing the impact of ITNs.

In our study, holes and hole size were not epidemiologically important for infection, nor was bioefficacy of the ITN measured against fully sensitive laboratory-reared mosquitoes. A study conducted in a neighboring district demonstrated phenotypic resistance of wild-caught mosquitoes to pyrethroids[36] and deltamethrin or permethrin-impregnated nets[37] even though the ITNs showed adequate bioefficacy against fully sensitive laboratory strains. It is possible that local wild-caught mosquitoes would have high levels of phenotypic resistance to pyrethroids. This is hinted at by the relationship of pesticide use to increased odds of malaria and needs to be explored further. If this is true, ITNs could perform well when tested against fully susceptible laboratory-reared strains but in reality offer reduced protection.

There was a strong correlation between malaria in the index child and the presence of other malaria-infected individuals in the home, whether or not they were symptomatic. Our observational study was not able to discern whether these infected family members increase the risk of infection for children in the home, or if we had simply succeeded in identifying households that had very high risk of infection amongst all their members. If the former, it provides a strong argument for ring testing and treatment[38, 39]. Molecular analyses of parasite genomes are required to try to determine the relationship between infections within a household.

Our study has several limitations. First, by recruiting cases in the hospital, our study focuses on children with disease (cases) compared to those without infection and was not designed to identify correlates of transition from infection to disease or progression to severe disease. However, by matching our controls to our cases on age, date, and village of residence, and by excluding controls who had recently been ill or taken antimalarials, we have likely eliminated potential confounding factors related to immunity and previous exposure. We were not able to evaluate the contribution of changes in mosquito behavior to malaria infection, nor did we investigate human behavioral factors that may have resulted in increased time exposed to outdoor or early biting. Other studies have attributed reduced ITN efficacy to outdoor biting or early biting by malaria vectors[40, 41] and human behavioral risk factors not solely related to use of ITNs while sleeping [42]. In addition, the small number of households enrolled in mosquito surveillance limited our ability to describe well the factors contributing to mosquito abundance. Finally, the net use information was self-reported and although we were able to strengthen our data by asking questions in multiple ways and supplementing with observed net hanging and use, reporting bias cannot be ruled out.

Other studies have individually investigated ITN ownership and coverage[43–48], correct use[49], or net condition[37, 50], and some have related these to household infection[51, 52]. Other studies have focused on vector resistance to insecticide [53, 54] and effects on vector feeding[55]. For the most part, these studies have isolated and investigated one or two important aspects of ITN efficacy or facilitating factors[56, 57]. Often they relate ITN variables to socioeconomic variables[58–61] but do not link them to epidemiologic outcomes. Here we present an integrated picture of ITN use in a community and allocate responsibility for persistent malaria burden to specific domains. We are able to measure the epidemiologic importance of specific problems associated with reduced efficacy of ITNs.

Our results highlight the gains to be made if ITNs are adopted as one component of integrated vector management. Facilitating factors that contribute to vector abundance and resistance to ITNs such as breeding site abundance, pesticide use, and household construction, were critical. It is imperative to move away from virtually exclusive reliance on ITNs for vector control. At the current ITN coverage level, we may be able to reduce malaria disease by 33% through implementing community-based environmental management. These results are supported by other studies[62, 63]. Such activities could significantly enhance the effectiveness of ITNs.

Enormous financial investments have been made in procuring and distributing ITNs. In order to maximize the impact of these investments, we need to understand factors beyond coverage. We need to develop an integrated picture of the context required for prevention success. Bungoma East holds important lessons for other malaria endemic communities but we also recognize that bottlenecks to effective prevention will most likely vary from place to place. In some places, vectors may begin to develop resistance perhaps due use of pyrethroids in local agriculture, in other places customs or sleeping arrangements may limit the use of ITNs, for example in nomadic communities or those that move during planting and harvesting seasons. Data must reflect local situations and it must be collected regularly to monitor changes, particularly in the case of emerging insecticide resistance. Furthermore, solutions to address bottlenecks vary by which step in the decay chain is affected. Local, timely information will allow solutions to be appropriate and customized.

## Supporting Information

S1 Table Unadjusted odds ratios for all variables tested in the model. (XLSX)
